# Face masks: benefits and risks during the COVID-19 crisis

**DOI:** 10.1186/s40001-020-00430-5

**Published:** 2020-08-12

**Authors:** Christiane Matuschek, Friedrich Moll, Heiner Fangerau, Johannes C. Fischer, Kurt Zänker, Martijn van Griensven, Marion Schneider, Detlef Kindgen-Milles, Wolfram Trudo Knoefel, Artur Lichtenberg, Balint Tamaskovics, Freddy Joel Djiepmo-Njanang, Wilfried Budach, Stefanie Corradini, Dieter Häussinger, Torsten Feldt, Björn Jensen, Rainer Pelka, Klaus Orth, Matthias Peiper, Olaf Grebe, Kitti Maas, Peter Arne Gerber, Alessia Pedoto, Edwin Bölke, Jan Haussmann

**Affiliations:** 1 Department of Radiation Oncology, Heinrich Heine University, Dusseldorf, Germany; 2 Department of the History, Philosophy and Ethics of Medicine, Heinrich Heine University, Medical Faculty, Dusseldorf, Germany; 3 Institute for Transplant Diagnostics and Cell Therapeutics, Heinrich Heine University, Dusseldorf, Germany; 4 Center for Biomedical Education and Research (ZBAF), University Witten/Herdecke, Witten, Germany; 5 Department cBITE, MERLN Institute for Technology-Inspired Regenerative Medicine, Maastricht University, Maastricht, The Netherlands; 6 Department of Experimental Anesthesiology, University of Ulm, Ulm, Germany; 7 Department of Anesthesiology and Intensive Care Medicine, Heinrich Heine University, Dusseldorf, Germany; 8 Department for General Visceral and Pediatric Surgery, Heinrich Heine University, Dusseldorf, Germany; 9 Department for Cardiac Surgery, Heinrich Heine University, Dusseldorf, Germany; 10 Department of Radiation Oncology, University Hospital, LMU Munich, Germany; 11 Department of Gastroenterology, Hepatology and Infectious Diseases, Heinrich Heine University, Dusseldorf, Germany; 12 Institute for Applied Statistics, Munich, Germany; 13 University of Hannover, Hannover, Germany; 14 Heinrich-Heine-University, Dusseldorf, Germany; 15 Department for Cardiology, Rhythmology and Intensive Care Medicine, Evangelic Hospital, Dusseldorf, Germany; 16 Department of Anesthesiology, Memorial Sloan Kettering Cancer Center, New York, NY USA

## Abstract

**Background:**

The German government has made it mandatory to wear respiratory masks covering mouth and nose (MNC) as an effective strategy to fight SARS-CoV-2 infections. In many countries, this directive has been extended on shopping malls or public transportation. The aim of this paper is to critically analyze the statutory regulation to wear protective masks during the COVID-19 crisis from a medical standpoint.

**Methods:**

We performed an extensive query of the most recent publications addressing the prevention of viral infections including the use of face masks in the community as a method to prevent the spread of the infection. We addressed the issues of practicability, professional use, and acceptability based on the community and the environment where the user resided.

**Results:**

Upon our critical review of the available literature, we found only weak evidence for wearing a face mask as an efficient hygienic tool to prevent the spread of a viral infection. However, the use of MNC seems to be linked to relevant protection during close contact scenarios by limiting pathogen-containing aerosol and liquid droplet dissemination. Importantly, we found evidence for significant respiratory compromise in patients with severe obstructive pulmonary disease, secondary to the development of hypercapnia. This could also happen in patients with lung infections, with or without SARS-CoV-2.

**Conclusion:**

Epidemiologists currently emphasize that wearing MNC will effectively interrupt airborne infections in the community. The government and the politicians have followed these recommendations and used them to both advise and, in some cases, mandate the general population to wear MNC in public locations. Overall, the results seem to suggest that there are some clinically relevant scenarios where the use of MNC necessitates more defined recommendations. Our critical evaluation of the literature both highlights the protective effects of certain types of face masks in defined risk groups, and emphasizes their potential risks.

## Introduction

The knowledge that the use of face masks delays the SARS-CoV-2 transmission is rapidly gaining popularity in the general population. Politicians need guidance on how masks should be used by the public to fight the COVID-19 pandemic crisis. In this review, we summarize the relevant literature on this topic.

“The surgical face mask has become a symbol of our times.”

On March 17th, 2020, this was the headline of an article in the New York Times on the role of face masks during the COVID-19 outbreak. Face masks have become a clothing accessory that is worn every day and everywhere. A variety of shapes, forms, and materials are being used and advertised to the point that in 2020 the business of producing and selling face masks was born.

In Germany, the government has ruled that wearing a face mask is obligatory to protect the population from any risks of airborne illness, according to the constitutional law [1] stating that “Protection must be easily provided to every citizen in the country.”

The aim of this paper is to analyze and critically discuss the regulations of some Federal States in Germany, which require protective masks in public to conform to similar regulations already in place in other countries.

Most masks covering the mouth are named mouth nose covering (MNC) according to the Robert Koch Institute (RKI; the German federal government agency and research institute responsible for disease control and prevention) and do not protect against respiratory and airborne infections. In the following review, the term “protective masks” will be used to describe any type of face mask.

## Face masks protecting from infections

**Fig. 1:**
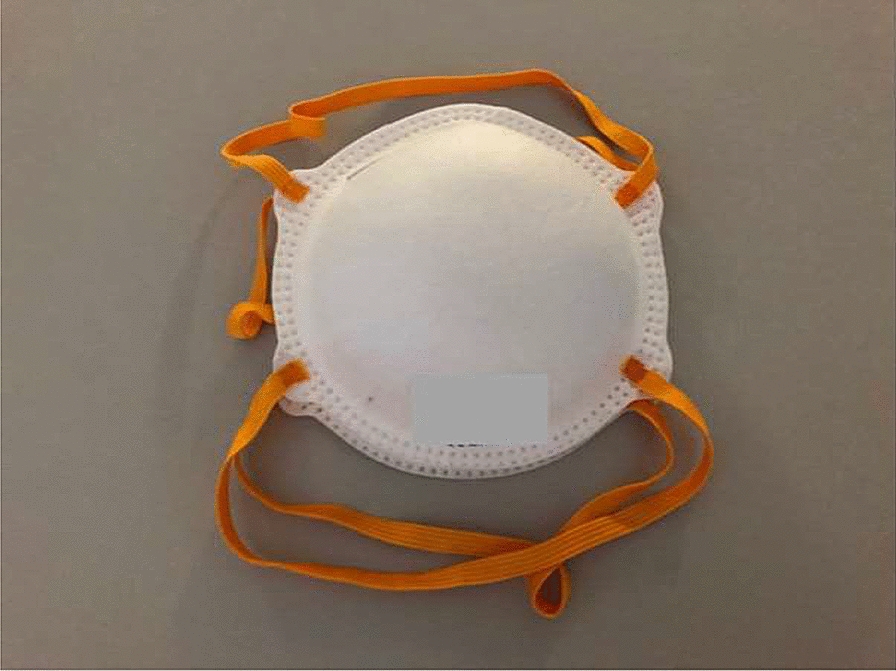
FFP (*filtering face piece)* mask without valve

**Fig. 2:**
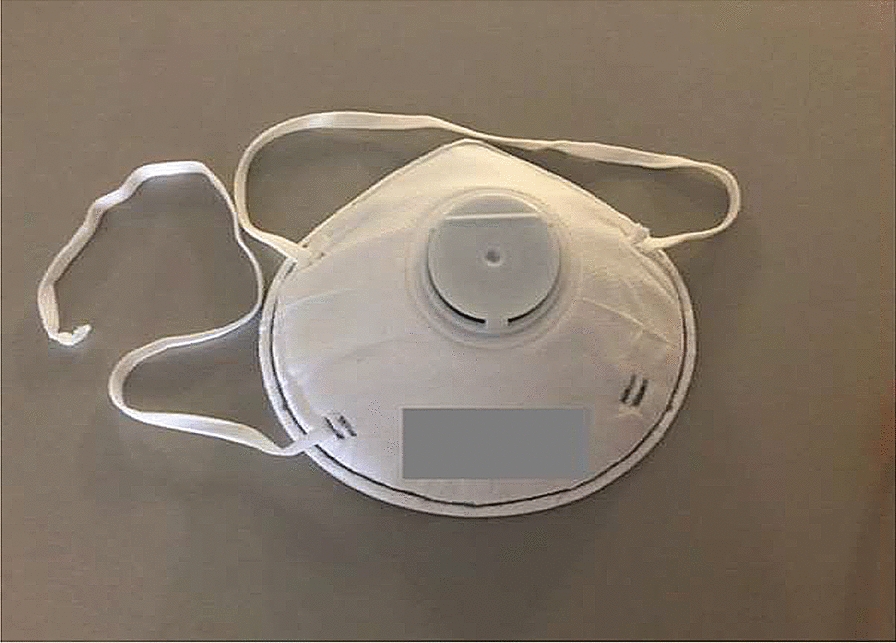
FFP (*filtering face piece)* mask with valve

**Fig. 3:**
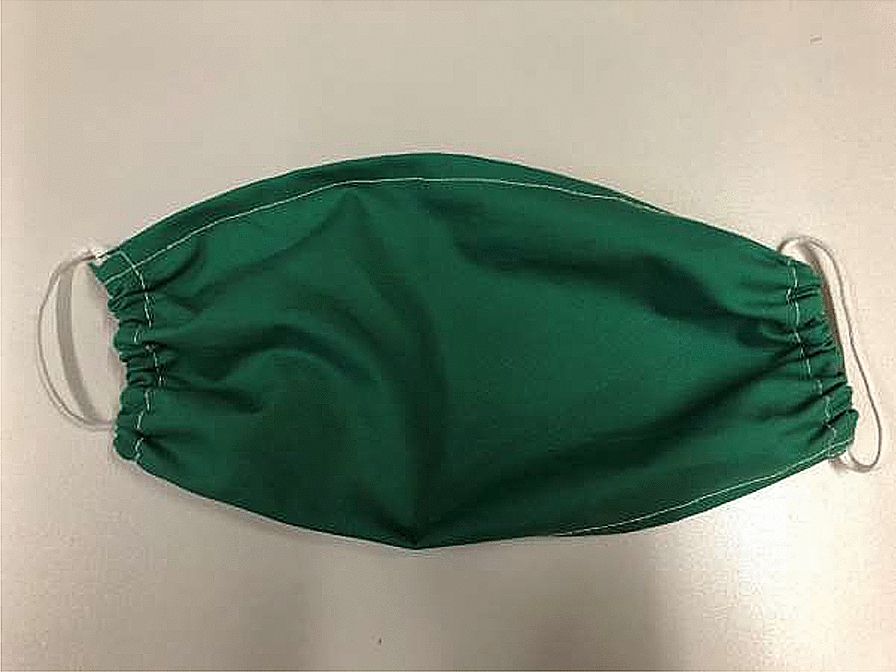
Homemade face mask for everyday use

**Fig. 4:**
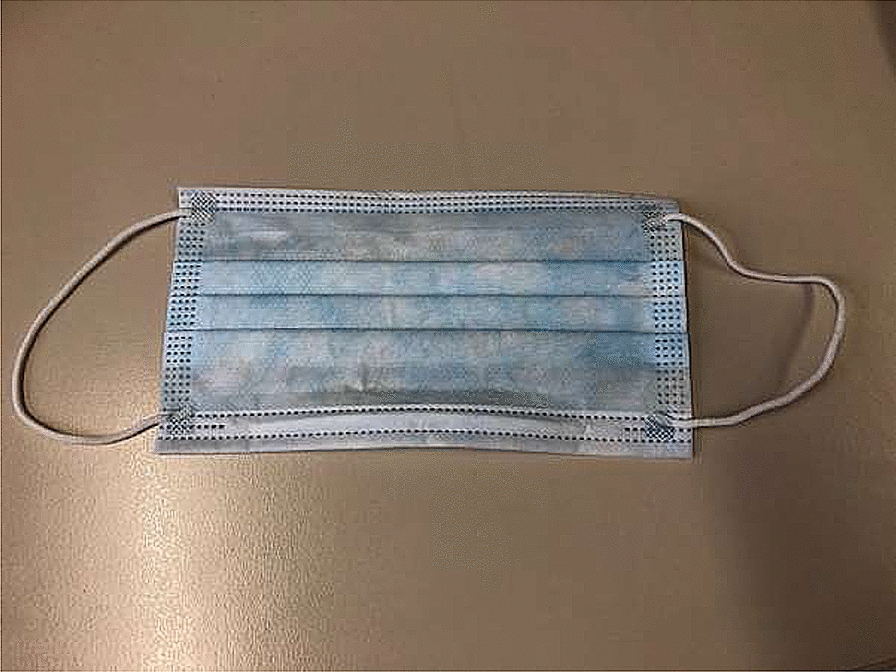
Surgical mask (MNP)

Respiratory masks (RM) are protective devices covering a part of the face. They are designed to protect both the person who wears them and the immediate environment from breathable pollutants (respiratory poisons or bacterial/viral pathogenic organisms). Different masks can be classified as I) *full masks* (normed following EN 136) and II) *half and quarter masks* (EN 140) (Figs. 1, 2, 3 and 4). While a *full mask* covers the whole face, a *half*-*mask* fits from under the chin to above the nose, a *quarter mask* fits from the top of the nose to the top of the chin. The breathing resistance varies proportionally to the density of the mask material.

**FFP masks** (*filtering face piece)* are classified as *half masks*. Their use is required to prevent the entry of pathogens through the airway and have the role of protecting both the wearer and the surrounding people. They are different from medical MNC, (often referred to as “surgical masks”), and from “self-made” masks for everyday use. MNCs and self-made masks are not “leak-proof” and do not provide complete respiratory protection since air can escape through them. FFP masks come without (Fig. 1) or with (Fig. 2) a valve. FFP (filtering face piece) masks with valves provide an air flow from the inside to the outside of the mask. FFP 1 masks are dust masks and mainly used for this purpose. They do not prevent COVID-19 infections. FFP1 masks are suitable for work environments in which only non-toxic dusts are found. FFP2 masks are suitable for work environments where there are pathogens and mutagens in the air composition.

In the context of SARS-CoV-2 the following types of masks are available (WHO, 2020):

1. Masks for everyday use (temporary masks made from fabric, etc.; Fig. 3): These masks grant no protection for the user from being infected. However, it is safe to assume there is a small risk reduction for droplet transmission, especially during exhalation, resulting in a reduction of potential viral spread. These masks should not be used in the health care system, but are commonly recommended for the general population for walking, shopping, or using public transportation.
2. MNP (= medical mouth–nose protection; Fig. 4): often referred to as a “surgical mask”. The industrial production of MNP abides to strict rules to provide protections against infection. The filtering capability is like the one for everyday use masks and they are intended to protect patients. They are approved for medical staff use, warrantying only patient-protection, specifically aimed against aerosols.
3. FFP2-mask (= face filtering piece)/N95-mask: FFP2-masks fulfil a set of stricter protective norms. They protect the person wearing them, as > 95% of particles and droplets are held back when inhaling. FFP2-masks also effectively protect the environment as long as there is no exhaling valve. In contrast, masks with an exhaling valve let exhaled air pass out unfiltered, with contamination of the immediate environment.
4. FFP3-mask: FFP3-masks protect the user even more effectively than FFP2, as > 99% of droplets and particles are filtered when inhaling. FFP3-masks also protect the environment in the absence of an exhaling valve.

**Fig. 5:**
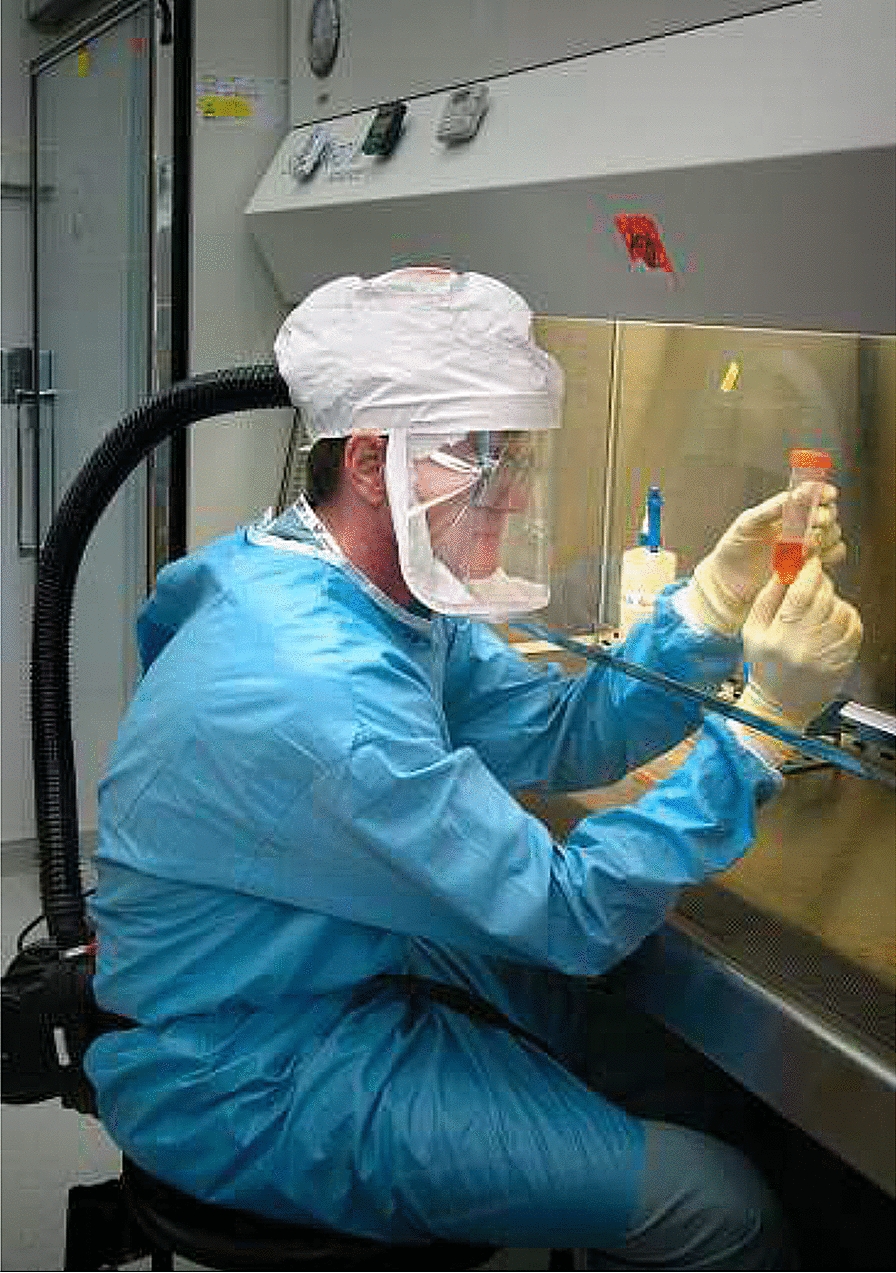
Full face mask in a level-3 biosafety lab (source: Wikipedia https://en.wikipedia.org/wiki/Face_masks_during_the_COVID-19_pandemic)

A full face mask in a level-3 biosafety lab is shown in Fig. 5.

The WHO states that the declared protective effect of these masks recommended during the SARS-CoV-2 pandemic can be severely reduced by their inappropriate use, such as improper donning or doffing, insufficient maintenance, long or repeated use of disposable masks, no dry cleaning of fabric masks, or using masks made of non-protective material [2].

During an epidemic/pandemic crisis every possible risk reduction strategy is useful. It is likely that the risk of infection and its severity depends on the viral load entering the body. This was the rationale for the Robert Koch Institute (RKI) to recommend the use of masks starting from March 2020. Specifically, they looked at the availability of the resources and tailored the supply to the risk of infection. Healthcare workers were considered essential workers at high risk of infection, therefore prioritized to the use of FFP2/3 masks, while MNC or masks for everyday use were to be made available for the general population.

## Current decree on wearing a mouth and nose covering

Due to the German Federalism, the Federal Minister of Health can only make health recommendations, which are then reinforced by the Infection Protection Act of the different Federal States. In the current situation of a pandemic crisis, nearly all measures are taken to prevent an exponential increase of new SARS-CoV-2 infections.

As of June 1st, 2020, the Netherlands considers the public use of protective masks unnecessary. This is based on the assumption that SARS-CoV-2 is only transmitted as a droplet infection via the nasopharynx pathway, which mostly occurs during coughing or sneezing. These droplets do not stay in the air, but rather drop to the ground within a 1.5 m radius if larger than 5 µm [3]. It has been postulated that for SARS-CoV-2—in contrast to other respiratory-driven infections—the droplets in the aerosols are of little relevance for a COVID-19 outbreak. Therefore, securing a 1.5-m social distance is assumed to be an essential and sufficient preventive measure. However, recent data published in 2020 using high-speed cameras show that small droplets of saliva and mucus can fly up to 8 m [4], requiring critical reconsideration of the above-mentioned assumption.

We conducted a Medline survey to scientifically justify this approach with the key words SARS-CoV-2, face masks, COVID-19, pandemic.

Leung and colleagues [5] screened more than 3000 individuals and identified 123 patients suffering from a viral respiratory infection. The viral load in the exhaled aerosol and droplets were different depending on the etiology of the infection, but was exponentially reduced by wearing surgical masks (cat. no. 62356, Kimberly-Clark). More viral particles were released through coughing. Generally, the authors reported a notably higher viral load in nose swabs compared to throat swabs. This data applied to influenza, corona, and rhino virus. No data are available for SARS-CoV-2 yet.

In general, droplets, and hence SARS-CoV-2, can be transferred via direct contact or smear transfection modality when the hands are contaminated from touching the nose or the face and then come in direct contact with others, e.g. by handshaking. For this reason, not only the “*cough etiquette*”, but regular and thorough handwashing are a significant and mandatory hygienic rule (6).

As a result of scientific data combined with daily routine, the RIVM (Rijksinstituut voor Volksgezondheid en Milieu, the Dutch equivalent of the RKI) has mandated to wear masks while using public transportation, due to the inability of maintaining enough protective distance, especially when riding during rush hour. This rule does not apply to other public spaces yet.

## Summarizing the arguments in favour of wearing a mask

- Wearing a mask in areas where sufficient distance is not feasible, such as public transportation, most likely reduces the spread of virus-loaded droplets and therefore the risk of transferring SARS-CoV-2.
- It is indisputable that infected patients can transfer SARS-CoV-2 to other people, starting few days before manifesting clinical symptoms or during the incubation period. However, there is no reliable data concerning the amount of virus particles that can be spread by an asymptomatic person, when keeping a minimum safe distance.

## Main arguments against wearing a mask

- If there is a limited supply of protective masks, they should be reserved for health care workers in hospitals and care facilities. This applies for surgical masks and for FFP2 and FFP3 masks.
- Masks give a false sense of security. The main role of MNC is the protection of people standing nearby. MNC do not protect the wearer.
- It is essential to wear the mask correctly. It must fit airtight to the skin, otherwise its effect is lost. Doffing of the mask needs to be properly done as well. The outside of the mask should not be touched. When supply is not an issue, surgical masks should be used only once.
- The lack of nonverbal communication when wearing a mask may make people feel insecure, disheartened or even psychologically troubled. This may be particularly true for people suffering from mental illness or hearing impairment.
- Breathing dampens the mask. If there is excessive moisture, the masks become airtight. Therefore, air is inhaled and exhaled unfiltered around the edges, losing the protective effect for both the wearer and the environment.
- If masks are not exchanged regularly (or washed properly when made of cloth), pathogens can accumulate in the mask. When improperly used, the risk of spreading the pathogen—including SARS-CoV-2—might be critically increased.

## Protective masks in context of rivalling concerns

In Germany, the COVID-19 pandemic has been more contained than in other European countries or even worldwide. However, we are not immune to this infection. It is imperative to implement any measure to control the spread of the infection, or at least the speed of diffusion to the population. It is important to make sure that the German health care system does not deplete its resources. Theoretically, we are affected by the scarcity of mask supply like other nations or countries. People who risk their health and even their lives need to be protected. There should be a fine balance when suggesting preventive measures, since reinforcing them indiscriminately may contribute to psychological discomfort, acts of violence, and financial strain.

## Available data

The summarized studies examine different types of masks focussing on FFP/N-95 masks. As expected, there are no scientific studies on economic and social consequences of wearing masks (Table 1).

**Table 1:** Most important publications

First author	Year	Recommendation
Li [8]	2008	N95 masks offer considerably better protection from influenza and SARS virus
Zhou [11]	2018	Protective effect for N95 masks for influenza and rhinovirus
Verbeek [13]	2020	Protecting the whole body is not superior to protecting different parts separately
Konda [12]	2020	Cotton masks have a high protective effect

In the following, the most important results are summarized.

### Study 1—PPE

Chia et al. (2005) [6] used a questionnaire to analyze the perception of doctors, nurses and other personnel on the role of PPE (= personal protective equipment) during the SARS-outbreak in Singapore over a period of 2 months in 2003. In summary, 32.5% of doctors, 48.7% of nurses and 77% of the administrative personnel thought that a simple MNP would be sufficient to prevent the SARS-infection. It was evident that even qualified staff did not have sufficient knowledge on the protective properties of face masks during a pandemic. This study highlights the importance of adequate communication, education and exchange of information in a timely fashion.

### Study 2—MNP masks

Lipp et al. (2005) [7] investigated the pattern of use and the protective effects of masks on wound infections using a questionnaire in two randomized studies. While the use of MNP was statistically beneficial in a smaller study (n = 200), the same recommendations were not valid when a larger cohort (n = 1250) was studied.

### Study 3—MNP vs. N95 valve masks

Li (2008) [8]: this study compared the protective effects of simple MNP with two different N95 masks with different valve systems. In contrast to the commonly available masks, this model had valves placed on the sides and was studied in an experimental setting with artificial droplets. All masks blocked the inside transmission of droplets from the front. The effectiveness of the regular MNP mask was only 95–97% when compared to the N95, which had a protective effect of 99%. Thus, N95 masks offer considerably better protection from influenza and SARS virus infections when compared to other mask types.

### Study 4—masks for everyday use

Rengasamy (2010) [9]: the protective effect of masks for everyday use made from different materials was tested against 20–1.000 nm particles with different velocities and compared to N95 masks. This study found marginal protective effects against exhaled particles. Specifically, depending on the material and dampness, 40–90% of aerosols were able to penetrate through these masks.

### Study 5—N95 vs. MNP

Smith et al. (2016) [10] analyzed all the available literature from 1990 to 2014, including 3 randomized controlled studies, one cohort study and 2 case–control studies comparing MNP vs N95 masks. Their meta-analyzis assessed: (a) the laboratory-proven infection rate, (b) influenza-related infections, and (c) work absence secondary to illness in employees. Their results indicated that the overall calculated risk assessment is not considerably improved using more sophisticated N95 masks.

### Study 6—N95

Zhou and colleagues (2018) [11] examined the role of various features on N95 masks, including valves for a more comfortable breathing, on the rate of infection. The endpoint was the retention of small particles of around 2.5 µm. The results revealed that the protective effect was sufficient against the examined viruses including influenza and rhinovirus.

### Study 7—masks for everyday use

Konda et al. (2020) [12] investigated the use of different materials on the effective filtration capabilities of masks for everyday use. They demonstrated that a combination of different materials such as cotton and silk, can be more effective than one material alone. Moreover, they revealed that densely woven cotton provides significantly more protection than cotton with looser weaves. A proper fit is particularly important to avoid leakage. The authors recommended the use of cotton masks that have a high protective effect and only little restriction when breathing.

### Study 8—meta-analysis comparing PPE partial vs. complete protection

Verbeek (2020) [13]: a recent meta-analysis investigating PPE (personal protection equipment) masks looked at 24 studies with a total of 2.278 participants. Fourteen studies were randomized, one was quasi-randomized and nine had no study design with randomization. Eight studies compared different PPE even though personal protective equipment included more than the mask. Six studies evaluated the quality of the protective equipment. 75% of these studies used a simulated exposure with fluorescent markers tagged on harmless microbes. They concluded that protecting the whole body is not superior to protecting different parts separately. Furthermore, proper donning and doffing protocols were more beneficial in preventing the spread of the disease. Both steps require proper training to be effective.

## Conclusion of the studies

Currently, most of the literature available on this topic is from experimental investigations. As expected, all the studies demonstrated an increase in protective effects in the following order: masks for everyday use–MNP–N95/FFP–PPE. Masks for everyday use can have a small protective effect for the wearer. MNP offers a greater protective effect since it was originally designed to decrease droplet elimination, therefore protecting the user’s surroundings. Unfortunately, due to ethical reasons, there is a lack of randomized controlled studies on the protective role of masks in the prevention of SARS-CoV-2 infections when compared to a control group with no masks. Since the Netherlands lack of a law to wear protection masks in public except for public transport since May 2020, it could serve as the control in future studies that compare the infection rates of different countries with different approaches to tackling the pandemic.

In 2016, Smith et al. [10] concluded that possible advantages of wearing a mask were difficult to apply to the social “day-to-day” situation. Konda et al. (12) highlighted the inability to discriminate between the protective effects of the mask on the environment, when worn by an infected person, versus the general protective effect within a given population. This would not have a significant health benefit if only a small percentage of individuals were infected. Only a study done in infected people with and without masks would allow a clear conclusion on the role of masks on the spread of the infection. Finally, a lesson learnt from the COVID pandemic shows significant educational gaps and lack of basic training that need to be addressed. The state should guarantee mask supply for everyone and educate on the proper use. Mass means of communication could be used for this purpose. A commercial broadcast before the daily news about the correct donning and doffing of the mouth and nose protection and its disinfection could reach a vast audience. In addition to public law, private and digital media, as well as healthcare providers such as doctors, pharmacists and nursing staff could also play an important role in education.

## Consequences of the use of protective masks on the wearer—pathophysiologic considerations

Wearing a mask has its own advantages and indisputable protective effects against infections. However, there are also potential risks and side effects that require attention. This specifically applies to the use in the general population.

From a medical standpoint, there is a theoretical possibility of an airflow obstruction when wearing a mask. A subjective feeling of strained breathing rarely occurs when wearing surgical masks. When wearing very dense masks without valves (N95/FFP2-3), breathing occurs against an air flow resistance. Theoretically, an increase in work of breathing can occur, especially during physical exertion.

Depending on the design, masks can increase the lung’s dead space. In extreme cases, carbon dioxide retention (hypercapnia) can occur with side effects. Only few investigations are available and addressing this medical problem. The available literature examined different types of N95 masks in the industrial setting in detail [14–16], and found relevant effects on the wearer. In this context, Kim et al. [17] studied the role of N95 masks on lung function and heart rate during low-to-moderate exercise/physical work load. Only healthy subjects seem to tolerate wearing such a mask. Studies conducted on employees in advanced stages of pregnancy showed a good tolerance for masks. The results of this study, even though specific to this population, are valuable for the daily use of MNP as a general mean of protection [18]. Finally, the role of N95/FFP-2 masks was tested in 97 patients with advanced COPD while undergoing a 6-min walk test. Seven patients did not tolerate the test and stopped prematurely. The respiratory rate, oxygen saturation and CO_2_ levels changed significantly while wearing N95/FFP2 masks. These results demonstrated the potential risks of wearing this type of mask in the presence of advanced COPD [19]. Their use should be recommended with caution in this patient population, a questionably relevant recommendation, since the use of these masks is limited to health care workers in direct contact with COVID patients. Finally, people with hearing impairment rely on lip reading to understand others. This is not possible when wearing a mask.

## Conclusion

Measures to prevent infections are necessary in the current pandemic. Face masks have been considered a first step to prevent and contain the spread of the disease. Different types of masks are available on the market for this purpose.

Simple masks covering mouth and nose are primarily used to prevent transmission by holding back droplets. This is useful when the recommended minimum distance of 1.5 m is not feasible. The masks provide only limited self-protection for its wearer and this is only when they are used properly.

High-quality FFP2/3 masks are a more reliable protection from infections. They should always be available for medical staff and people at risk. When used by the general population, specific groups at risk for complications related to the mask use should be educated on what to expect. For example, patients with severe COPD can experience a deterioration of their respiratory parameters. Therefore, patients must be individually educated by their general practitioner about the risk of wearing MNC.

Finally, it is imperative that the user is educated on the different types of masks available, how and when to wear them and, above all, how to handle them correctly, similar to the safety instructions given before take off in an aircraft.

Our results are consistent with the ones recently reported by Chu et al. in Lancet [20]. These publications will help guide the decisions of politicians and caregivers on when and where to use the available tools to fight a viral pandemic.

## Data Availability

All data and materials can be accessed via CM and FM.

## Abbreviations

COPD: Chronic obstructive pulmonary disease
COVID-19: Coronavirus disease 2019
EN: European normalization
FFP: Filtering face piece
MNC: Masks covering mouth and nose
MNP: Medical mouth–nose protection
N95: Masks filtering > 95% of particles and droplets
PPE: Personal protective equipment
RIVM: Rijksinstituut voor Volksgezondheid en Milieu the Dutch federal government agency and research institute responsible for disease control and prevention
RKI: Robert Koch Institute the German federal government agency and research institute responsible for disease control and prevention
RM: Respiratory masks
SARS: Severe acute respiratory syndrome
SARS-CoV-2: Severe acute respiratory syndrome coronavirus 2
WHO: World Health Organization
